# RNA N6-Methyladenosine (m6A) Methyltransferase-like 3 Facilitates Tumorigenesis and Cisplatin Resistance of Arecoline-Exposed Oral Carcinoma

**DOI:** 10.3390/cells11223605

**Published:** 2022-11-14

**Authors:** Chuang Wang, Chamila Kadigamuwa, Songlv Wu, Yijun Gao, Wuya Chen, Yangcong Gu, Shengli Wang, Xia Li

**Affiliations:** 1 Department of Oral Medicine, Foshan Stomatological Hospital, Medical College of Foshan University, Foshan 528000, China; 2 Department of Chemistry, University of Kelaniya, Kelaniya 11600, Sri Lanka; 3 Department of Stomatology, The Second Xiangya Hospital, Central South University, Changsha 410008, China; 4 Department of Oral Maxillofacial Surgery, Foshan Stomatological Hospital, Medical College of Foshan University, Foshan 528000, China

**Keywords:** oral squamous cell carcinoma, arecoline, methyltransferase-like 3, cisplatin resistance, tumorigenesis

## Abstract

Background: Arecoline is known as the main active carcinogen found in areca nut extract that drives the pathological progression of oral squamous cell carcinoma (OSCC). Studies have revealed that dysregulation of RNA N6-methyladenosine (m6A) methyltransferase components is intimately linked to cancer initiation and progression, including oral cancer. Methods: The arecoline-induced dysregulated methyltransferase-like 3 (METTL3) gene was identified using RNA-seq transcriptome assay. Using in vitro and in vivo models, the biological roles of METTL3 in arecoline-transformed oral cancer were examined. Results: We found that METTL3 was markedly elevated in arecoline-exposed OSCC cell lines and OSCC tissues of areca nut chewers. We identified that hypoxia-inducible factor 1-alpha (HIF-1α) stimulated METTL3 expression at the transcriptional level and further proved that METTL3-MYC-HIF-1α formed a positive autoregulation loop in arecoline-transformed OSCC cells. Subsequently, we manifested that METTL3 depletion profoundly reduced cell proliferation, cell migration, oncogenicity, and cisplatin resistance of arecoline-exposed OSCC cells. Conclusions: Developing novel strategies to target METTL3 may be a potential way to treat OSCC patients, particularly those with areca nut chewing history and receiving cisplatin treatment.

## 1. Introduction

Oral cancer, which is highly prevalent in Southeast Asian countries, is the sixth most frequent malignancy worldwide. According to the World Health Organization (WHO), more than 657,000 new cases of oral cavity and pharynx cancer are identified each year, with more than 330,000 deaths occurring worldwide [1,2]. Oral cancer is especially deadly as it typically presents without pain or symptom in the early stages, and the majority of cases are detected in the later stages [3]. By the late stages, oral cancer usually spreads to other organs, including the cervical lymph nodes, skin, lung, liver, etc. Oral cancer is fatal, with a five-year survival rate of roughly 50% [4]. Hence, identifying essential genes involved in the development of oral cancer is vital for designing innovative targeting techniques for treating oral cancer.

Tobacco, alcohol, and areca nuts are carcinogens that contribute to the onset, development, and progression of oral cancer [5]. Numerous studies have established a strong link between areca nut exposure and oral cancer [6]. Areca nut chewing is practiced by approximately 85% of oral cancer patients in Taiwan [7]. Chewers of areca nuts have a considerably increased chance of developing oral cancer than nonchewers. In addition, oral cancer patients who frequently consumed areca nuts exhibited a considerably poorer five-year survival rate. As a result, the International Agency for Research on Cancer (IARC) and WHO have categorized areca nut/betel nut as category I carcinogens to human. The most common alkaloid found in areca nut extract, arecoline, is thought to be the main active carcinogen responsible for the pathological initiation and development of oral cancer [8]. Arecoline has long been linked to the initiation, development, and progression of oral cancer due to its mutagenic and genotoxic effects [9]. However, the complex effects of arecoline on oral cancer’s progression as well as the underlying molecular pathways remain not completely understood.

Dysregulation of tumor-suppressing genes and oncogenes is a hallmark of cancer. DNA modifications have been identified as an important epigenetic regulation mechanism that contributes to cancer initiation and progression [10]. RNA N6-methyladenosine (m6A) modifications have emerged as new frontiers in cancer biology in recent years [11]. m6A methyltransferase components are able to mediate m6A modification, which has been linked to the regulation of different cellular functions by altering targeted mRNA splicing, translation efficiency, and stability. The m6A methyltransferase components primarily comprise three types of enzymes: “readers” (e.g., YT521-B homology (YTH) domain family proteins), “writers” (e.g., methyltransferase-like 3 (METTL3)), and “erasers” (e.g., alkB homologue 5 (ALKBH5)) [12,13]. Recent research has shown that several of these genes are implicated in the development of oral cancer [12,14,15]. The m6A modification gene’s role in arecoline-mediated oral cancer development and transformation, on the other hand, remains elusive.

In this investigation, we aimed to identify a critical m6A modification gene that is important for the development of arecoline-induced OSCC. A deeper understanding of the biological function of m6A modification gene in arecoline-promoted oral cancer development might aid in the development of innovative strategies to cope with arecoline-induced OSCC.

## 2. Materials and Methods

### 2.1. Human Sample Collection

A total of 20 oral mucosa tissues and 66 OSCC tissues were collected in the Foshan Stomatology Hospital and Chenzhou No. 1 People’s Hospital from 2015 to 2022. All patients gave consent for the research. Among the 66 OSCC tissues, 29 were collected from individuals who chewed areca nuts and 37 OSCC tissues were acquired from individuals who had never consumed areca nuts. All collected human tissues were stained with hematoxylin and eosin and analyzed separately by two pathologists at Foshan Stomatology Hospital to confirm that each collected OSCC tissue contained greater than 80% tumor tissues.

### 2.2. Cell Culture, Cell Transfection, and Lentiviral Transduction

OSCC cell lines (SCC25, and CAL27) were purchased from the American Type Culture Collection (ATCC) (Manassas, VA, USA). CAL27 and SCC25 were grown in recommended complete DMEM medium and were kept at 37 °C in an incubator with CO_2_ (5 %).

Chronic arecoline-exposed CAL27 and SCC25 cell lines were established as previously described [12]. Briefly, OSCC cell lines were cultivated in full culture media supplemented with arecoline (1 μg/mL) or DMSO as a control. The medium containing arecoline was replenished every two days. The cells were passaged around 23 times throughout the arecoline treatment, which lasted 90 days.

Cell transfection or lentiviral transduction was used to achieve gene overexpression or knockdown. ThermoFisher Scientific supplied siRNAs targeting METTL3 (s32143), MYC (s9129), HIF-1α (s6539), or nonsense control (NC) (4390843). Millipore Sigma provided lentiviruses with shRNA targeting human METTL3 (TRCN0000034714), human MYC (TRCN0000039640), and shRNA control (SHC002). GeneCopoeia (Rockville, MD, USA) provided lentivirus with open reading frame cDNA of METTL3 (ULP-A8711-Lv105-A00), HIF-1α (ULP-T0096-Lv105-A00), MYC (LPP-B0255-Lv105-050), and negative control (LP105-025). Lentiviral particles transduced targeted cells at a multiplicity of infection (MOI) of 3.

### 2.3. Immunohistochemistry (IHC) Analysis

IHC staining was performed on paraffin-embedded tissues on 4 μm thick slides. Sections were baked in xylene, followed by deparaffinization and rehybridization using a graded ethanol series before being treated with antigen retrieval buffer. The slides were soaked in hydrogen peroxide (0.3%) for 10 min. After chilling for 20 min, slices covered with primary antibody were kept in a cold room overnight. After rinses with phosphate-buffered saline, sections were stained with appropriate secondary antibody and then observed under a microscope. Anti-METTL3 antibody ([EPR18810] (ab195352), 1:100 dilution) and anti-HIF-1α antibody ([EPR18810] (ab195352), 1:100 dilution) used for IHC staining were from Abcam (Boston, MA, USA).

### 2.4. Luciferase Reporter Assay

The PCR products of either the wild-type METTL3 5′-flanking region encompassing two putative binding sites or a 5′-flanking region with mutations in one or both putative binding sites were digested with Bgl II and Kpn I restriction enzymes. After purification, the PCR products were ligated into the luciferase reporter plasmid (pGL3-basic vector). These plasmids were sequenced to verify the sequence of the insert in each plasmid. To perform luciferase reporter assay, 293T cells were either transduced with lentivirus HIF-1α or lentivirus control or transfected with si HIF-1α or si control. The cells were cotransfected with the indicated luciferase reporter plasmid and pRT-TK vector. Twenty-four hours post-transfection, a dual-luciferase reporter assay kit was used for cellular sample preparation. The luciferase levels in each cellular sample were measured using a GloMax^®^ 20/20 Luminometer. The primer sequences used for luciferase reporter construction are listed in Table S1.

### 2.5. RT-qPCR

Tissue samples were quickly frozen using liquid N_2_, crushed into tiny pieces (<1 mm^3^), and soaked in TRIzol reagent (Invitrogen, CA, USA). OSCC cell pellets were immersed in TRIzol reagent. After RNA extraction and purification, the SuperScript III First-Strand Synthesis Kit was utilized to reverse-transcribe RNA (1 µg) into cDNA. The cDNA was mixed with target gene primers and SYBR™ Green PCR Master Mix (ThermoFisher Scientific, San Jose, CA, USA). Quantitative PCR (qPCR) assay was executed using an ABI Prism 7500 System (ThermoFisher Scientific). GAPDH was employed as the internal control. The 2−ΔΔCt method was applied to calculate the gene expression level, and the data were further normalized to GAPDH to obtain a relative gene expression level. The primer sequences used for qPCR are summarized in Table S1.

### 2.6. Chromatin Immunoprecipitation (ChIP)–qPCR Assay

The ChIP–qPCR test was carried out following the instructions provided by Pierce™ Agarose ChIP Kit (ThermoFisher Scientific, CA, USA). Briefly, cells were crosslinked in formaldehyde for 20 min and then stopped using stop buffer. After fixation, quenching, and washing the crosslinked cell pellet, the chromatin was extracted and sheared using the PIXUL™ Multi-Sample Sonicator (Active Motif, Carlsbad, CA, USA). The immunoprecipitation was performed using 3 μg of anti-HIF-1α antibody (ab195352, Abcam, UK) or control IgG (Abcam, Cambridge, MA, USA). After that, ChIP-qualified protein G agarose beads were used to retrieve antibody–antigen complexes. A DNA column elution solution was used to elute the DNA. qPCR analysis was performed on the isolated DNA.

### 2.7. Western Blotting

OSCC cell pellets were lysed in RIPA buffer supplemented with phosphatase and protease inhibitors (Pierce, ThermoFisher Scientific, Rockford, IL, USA). The protein samples were loaded on a Western blotting gel and separated by SDS-PAGE. After that, the proteins were transferred onto a PVDF membrane. The membrane was treated with primary antibodies overnight in a cold room and subsequently incubated with second antibodies. Using chemiluminescent substrates (Pierce), protein signals were developed and recorded using the Biorad Gel Doc XR Imaging System (Biorad, Hercules, CA, USA). Anti-MYC antibody (E5Q6W) (18583S, 1:1000 dilution) and Anti-GAPDH antibody (5174S, 1:5000 dilution) were from Cell Signaling Technology (Danvers, MA, USA). Anti-METTL3 antibody (ab195352, 1:1000 dilution) and anti-HIF-1α (ab1, 1:500 dilution) were from Abcam (Cambridge, UK).

### 2.8. Cell Proliferation Assay and Cell Migration Assay

The cell proliferation experiment was carried out using the cell counting kit-8 (CCK8). In a 96-well plate, cells were planted in triplicate. Following incubation at the desired time periods, WST-8 solution was supplemented, and the plate was kept at 37 °C in an incubator for around 3–4 h. The absorbance (450 nm) of each sample was determined employing a microplate reader.

OSCC cells were seeded in 8 μm pore size Transwell inserts (ThermoFisher Scientific, Rochester, NY, USA) containing serum-free medium. Then, these inserts were placed into the well of a 24-well culture plate filled with 0.5 mL complete medium. After being cultured for one day, the cells in the upper layer of inserts were scratched away using a swab. The migrated cells on the lower side of inserts were washed and fixed with paraformaldehyde. Following that, 0.5% crystal violet was used to stain the cells, and the cells were counted under a microscope.

### 2.9. ELISA

ELISA kits obtained from Raybiotech (Peachtree Corners, GA, USA) were utilized to assess the expression levels of IFN-γ (Kit Code: ELH-IFN-γ-1), IL-2 (Kit Code: ELH-IL-2-1), TNF-α (Kit Code: ELH-TNFα-1), GM-CSF (Kit Code: ELH-GM-CSF-1), IL-10 (Kit Code: ELH-TNFα-1), TGF-β (Kit Code: ELH-TGF-β-1), IL-17 (Kit Code: ELH-IL-17-1), and IL-4 (Kit Code: ELH-IL-4-1). Briefly, using sample diluent buffer, the tumor lysate samples were diluted to a lysate solution with a concentration of protein (1 mg) per original lysate (1 mL). Following the ELISA kit’s instruction, the lysate solution (100 μL) was dropped into the wells of the ELISA plate with precoated antibody. After incubating overnight, the plate was washed and further incubated with secondary antibody. After being treated with streptavidin solution, TMB substrate reagent was added into the wells to develop the signal. A microplate reader set at 450 nm was used to measure each well’s absorbance.

### 2.10. Mouse Tumorigenesis and Treatment

A total of 40 BALB/c strain nude mice aged around 6–8 weeks were purchased from the Guangdong Medical Science Experiment Center (Guangzhou, Guangdong, China). Mice were kept in an animal facility that was specifically pathogen-free. Subcutaneous injections of either CAL27-shRNA or CAL27-A-shMETTL3 were administered to mice. The division of the tumor-bearing mice into four groups (*n* = 10 for each group) was carried out at random a few days after the tumor was implanted. The animal study design was as follows. Groups 1 and 2: CAL-27-shRNA tumor-bearing mice received intraperitoneal (i.p.) injections of PBS (Group 1) or cisplatin 4 mg/kg (Group 2) twice per week. Groups 3 and 4: CAL-27-shMETTL3 tumor-bearing mice received i.p. injections of PBS (Group 3) or cisplatin 4 mg/kg (Group 4) twice per week. Starting at day 10 after tumor implantation, tumor volumes were assessed every three days. The formula used to determine tumor volumes was V = 1/2 (length × width^2^). Mice were euthanized 49 days after the tumor was implanted. Weight measurements were taken of the tumor tissues.

### 2.11. Statistical Analyses

The statistical analysis program GraphPad Prism (V.7) was used for statistical analyses. Student’s *t*-test results were utilized to compare the significance of the two groups. The Pearson correlation method was used to investigate the relationship between METTL3 and HIF-1 expression in OSCC tissues. The Mann–Whitney *U*-test and the Kruskal–Wallis test were used to compare differences between two and three groups, respectively, to analyze the correlations between METTL3 expression levels and clinicopathologic parameters. Statistical significance was defined as a *p* value < 0.05.

## 3. Results

### 3.1. MTTL3 Is Elevated in Arecoline-Exposed OSCC Cells and Tissues

**Figure 1:**
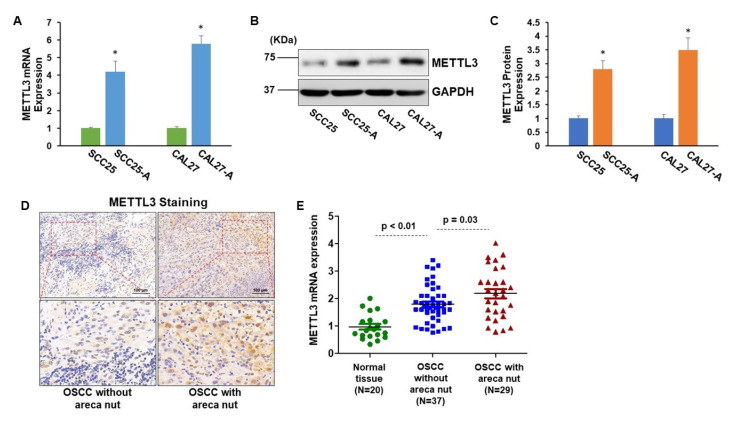
METTL3 is significantly elevated in arecoline-exposed OSCC cell lines and tissues. (**A**) The mRNA levels of METTL3 in SCC25, SCC25-A, CAL27, and CAL27-A were measured by RT-qPCR assay. (**B**,**C**) The expression levels of METTL3 in CAL27, SCC25, CAL27-A, and SCC25-A cells were assessed by Western blotting assay. The signal was quantified by Image J. (**D**,**E**) The METTL3 levels in normal oral tissues (*n* = 20), OSCC without areca nut (*n* = 37), and OSCC with areca nut (*n* = 29) were tested using IHC staining (**D**) and RT-qPCR assay (**E**). * *p* < 0.05 compared to the control group.

Our recent papers showed that OSCC cell lines that had undergone chronic low-dose arecoline transformation (designated as SCC25-A and CAL27-A) had greater tumorigenic effects than the parental OSCC cell lines (SCC25 and CAL27) [12]. The RNA-seq transcriptome results revealed that METTL3 gene was among the significantly (fold change > 2 and *p* < 0.05) elevated genes when comparing CAL27-A to CAL27 (Figure S1) [15]. To further confirm this result, the protein and mRNA levels of METTL3 in CAL27, CAL27-A, SCC25, and SCC25-A were measured. As illustrated in Figure 1A–C, the METTL3 mRNA and protein levels were substantially enhanced in CAL27-A and SCC25-A compared to their counterparts (CAL27 and SCC25) (Figure 1A–C). Moreover, we examined METTL3 expression in 20 oral mucosa tissues obtained from donors without areca nut exposure and 66 OSCC tumor specimens from OSCC patients, 29 of whom chewed areca nuts. As shown in Figure 1D,E, compared to OSCC specimens without areca nut exposure, METTL3 mRNA and protein levels were considerably greater in OSCC specimens with areca nut exposure, and METTL3 levels were noticeably higher in OSCC tissues than in healthy tissues (Figure 1D,E). More significantly, the correlation assessment of METTL3 levels with OSCC patients’ clinicopathologic parameters disclosed that high METTL3 levels were positively associated with local recurrence or distant metastasis and areca nut chewing habits but not with other clinical–pathological factors, including gender, age, tumor stage, or nodal stage (Table 1). These results suggest that METTL3 might be involved in arecoline-transformed OSCC malignancy.

### 3.2. HIF-1α Regulates METTL3 Expression

**Figure 2:**
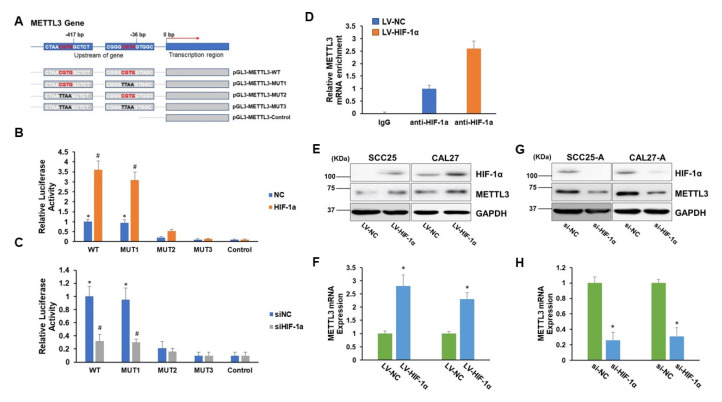
METTL3 is regulated by HIF-1α. (**A**) A schema of a variety of pGL3-METTL3 plasmids (WT, MUT1, MUT2, MUT3, or control). (**B**,**C**) 293T cells were transduced with lentiviral particles carrying HIF-1α or control (NC) (**B**) or transfected with siRNA targeting HIF-1α (siHIF-1α) or control (siNC) (**C**). Then, the cells were cotransfected with one of the pGL3-METTL3 plasmids (WT, MUT1, MUT2, MUT3, or control) along with pRT-TK vectors, which served as an indicator for transfection efficiency. The luciferase levels were de-termined 24 h after transfection. * *p* < 0.05 compared to the control group. # *p* < 0.05 HIF-1α group compared to the NC group or siHIF-1α group compared to the siNC group. (**D**) CAL27 cells were transduced with lentiviral particles (LV-HIF-1α or LV-NC). Chromatin immunoprecipitation analyses were executed with an anti-HIF-1α antibody or IgG followed by qPCR analyses of METTL3 gene. (**E**,**F**) CAL27, and SCC25 were transduced with lentiviral particles (LV-HIF-1α or LV-NC). The protein levels of HIF-1α, METTL3, and DAPDH were determined by Western blotting assay (**E**), and the METTL3 mRNA levels were tested by RT-qPCR assay (**F**). (**G**,**H**) CAL27-A and SCC25-A were transfected with siHIF-1α or siNC. The protein levels of HIF-1α, METTL3, and GAPDH were determined by Western blotting assay (**G**), and the METTL3 mRNA levels were tested by RT-qPCR assay (**H**). * *p* < 0.05 LV-HIF-1α group compared to the LV-NC group or siHIF-1α group compared to the siNC group.

To identify transcriptional regulatory sequences and potential transcription factors regulating METTL3 expression, we examined the 5′-flanking region from 2 kbp upstream of the transcription start site of METTL3 gene using genomatix (https://www.genomatix.de/solutions/genomatix-genome-analyzer.html, accessed on 10 October 2022). Of note, we identified two HIF-1α binding motifs (CGTG) located in the 5′-promoter region (−421/−417 and −40/−36) of the METTL3 gene (Figure 2A). To prove this discovery, we created a number of luciferase reporter plasmids with either the wild-type (WT) METTL3 5′-flanking region encompassing two putative binding sites or 5′-flanking region with mutations in one or both putative binding sites. The empty luciferase reporter plasmid without sequence insertion was used as a negative control (Figure 2A). As shown in Figure 2B,C, the plasmids with the wild-type (−421/−417) region (pGL3-METTL3-WT and pGL3-METTL3-MUT1), but not with the (−40/−36) region (pGL3-METTL3-MUT2), exhibited significantly elevated luciferase activities compared to the control. Forced expression of HIF-1α further increased, while depletion of HIF-1α reduced the luciferase activities of pGL3-METTL3-WT and pGL3-METTL3-MUT1 (Figure 2B,C). These results imply that HIF-1α might positively regulate METTL3 expression by binding to the (−421/−417) region. Furthermore, the chromatin immunoprecipitation (ChIP) assay showed that METTL3 DNA sequence containing the (−421/−417) region was significantly enriched in the anti-HIF-1α antibody immunoprecipitated complex (Figure 2D). Moreover, overexpression of HIF-1α greatly enhanced METTL3 levels in SCC25 and CAL27, whereas suppression of HIF-1α markedly reduced METTL3 mRNA and protein levels in SCC25-A and CAL27-A (Figure 2E–H). Collectively, these results indicate that the HIF-1α transcription factor binding sequence (CGTG, −421/−417) is the positive regulatory element of METTL3.

### 3.3. HIF-1α is Enhanced in Arecoline-Exposed OSCC Cells and Tissues

**Figure 3:**
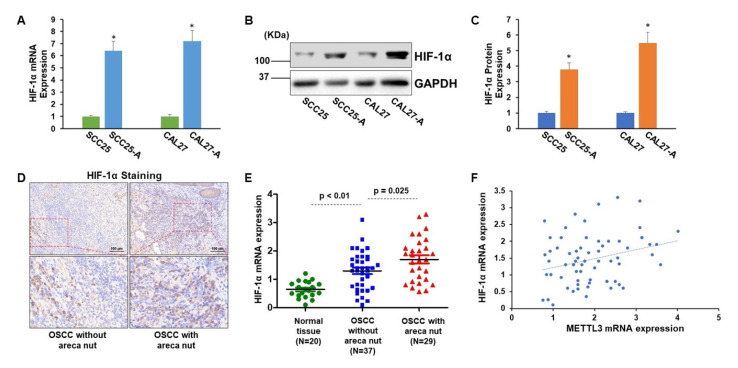
HIF-1α is markedly enhanced in arecoline-transformed OSCC cell lines and tissues. (**A**) The HIF-1α mRNA levels in CAL27, SCC25, CAL27-A, and SCC25-A were tested. (**B**,**C**) The HIF-1α protein levels in CAL27, SCC25, CAL27-A, and SCC25-A cells were measured (**B**) and quantified by Image J (**C**). (**D**,**E**) The HIF-1α levels in normal oral tissues (*n* = 20), OSCC without areca nut (*n* = 37), and OSCC with areca nut (*n* = 29) were identified using IHC staining (**D**) and RT-qPCR assay (**E**). * *p* < 0.05 compared to the control group. (**F**) Pearson correlation method was utilized to assess the association between HIF-1α and METTL3 in OSCC tissues. * *p* < 0.05 compared to the control group or as indicated.

Next, we aimed to study the expression profile of HIF-1α in OSCC cells with or without arecoline exposure and in OSCC tissues with or without areca nut exposure. As presented in Figure 3A–C, the HIF-1α expression levels were significantly upregulated in CAL27-A and SCC25-A compared to CAL27 and SCC25, respectively, implying that long-term arecoline exposure may induce HIF-1α expression (Figure 3A–C). Indeed, by examining HIF-1α expression in OSCC tissues, we found that HIF-1 levels in OSCC tissues exposed to areca nuts were significantly greater than in OSCC tissues not exposed to areca nuts (Figure 3D,E). Furthermore, the HIF-1α levels were positively correlated with METTL3 levels in OSCC specimens (*p* = 0.024, *n* = 66) (Figure 3F). In addition to indicating that exposure to arecoline/areca nuts can increase HIF-1α expression, these findings provide indirect evidence for METTL3 expression being regulated by HIF-1α.

### 3.4. METTL3-MYC- HIF-1α Forms a Positive Autoregulation Loop

**Figure 4:**
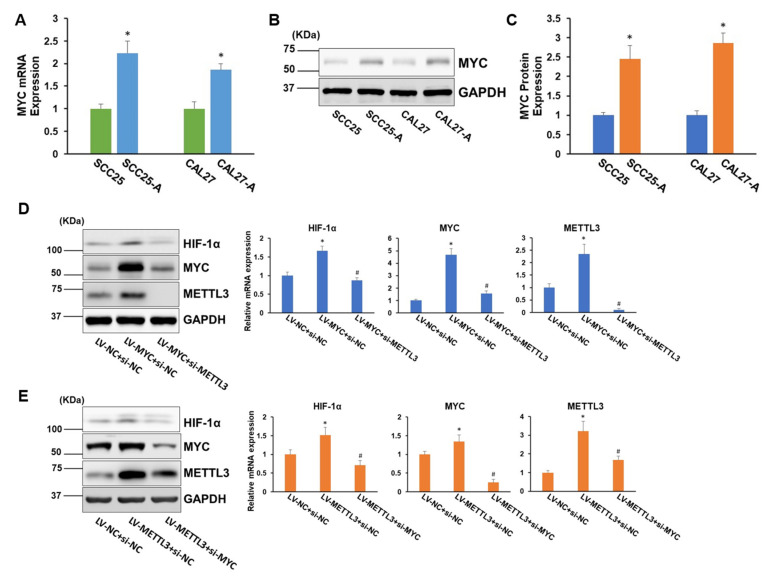
A positive autoregulation loop is formed between METTL3, MYC, and HIF-1α. (**A**) The mRNA levels of MYC in CAL27, SCC25, CAL27-A, and SCC25-A were measured. (**B**,**C**) The MYC protein levels in CAL27, SCC25, CAL27-A, and SCC25-A cells were tested (**B**) and quantified by Image J (**C**). (**D**) CAL-27 cells were transduced with lentiviruses carrying MYC cDNA (LV-MYC) or NC (LV-NC). Then, cells were transfected with siRNA targeting METTL3 (si-METTL3) or NC (si-NC). The protein and mRNA levels of HIF-1α, MYC, and METTL3 were measured. (**E**) CAL-27 cells were transduced with LV-METTL3 or LV-NC. Then, cells were transfected with siRNA targeting MYC (si-MYC) or NC (si-NC). The mRNA and protein levels of HIF-1α, MYC, and METTL3 were measured. * *p* < 0.05 compared to the control group. # *p* < 0.05 LV-MYC + si-METTL3 compared to LV-MYC + si-NC or LV-METTL3 + si-MYC compared to LV-METTL3 + si-NC.

We sought to investigate how arecoline affects HIF-1α expression in OSCC cells. Arecoline-induced MYC upregulation has been reported previously [16], and MYC is known to enhance HIF-1α accumulation via stabilizing HIF-1α in both normoxic and hypoxic conditions [17,18]. It is logical to believe that arecoline can modulate HIF-1α expression by mediating MYC activities. Intriguingly, METTL3 has been shown to increase MYC mRNA and protein levels via boosting MYC mRNA transcript m6A levels [19,20]. Based on these inspiring facts, we hypothesized that METTL3-MYC-HIF-1α may form a positive autoregulation loop under arecoline treatment. To prove this hypothesis, we first confirmed that MYC levels were notably upregulated in CAL27-A and SCC25-A compared to CAL27 and SCC25, respectively (Figure 4A–C). Furthermore, overexpression of MYC led to a significant increase in HIF-1α mRNA and protein levels. Depletion of METTL3 resulted in reduced MYC levels and further abolished the promotional effect of MYC-induced HIF-1α expression (Figure 4D). On the contrary, forced expression of METTL3 enhanced MYC levels and subsequently increased HIF-1α expression. The boosting effect of METTL3 upregulation on HIF-1α expression was abrogated by knockdown of MYC (Figure 4E). Collectively, these results confirmed the existence of a positive autoregulation loop among METTL3-MYC-HIF-1α.

### 3.5. Knocking down METTL3 Sensitized Arecoline-Transformed OSCC Cells to Cisplatin-Induced Cytotoxicity

**Figure 5:**
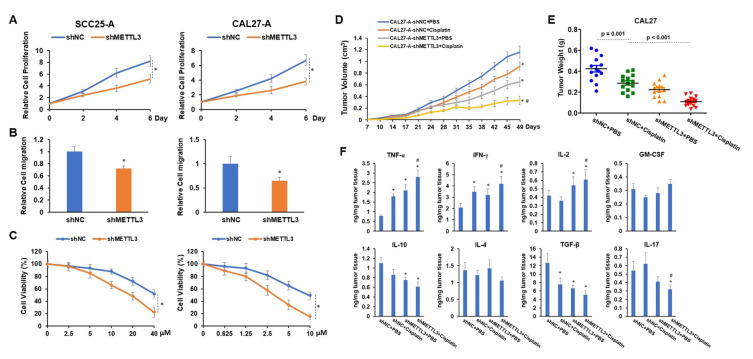
The proliferation, migration, tumor development, and cisplatin resistance of OSCC cells are all decreased when METTL3 is knocked down. Lentiviruses carrying shNC or shMETTL3 were used to infect SCC25-A and CAL27-A cells (referred to as SCC25-A-shNC, SCC25-A-shMETTL3, CAL27-A-shNC, and CAL27-A-shMETTL3). (**A**,**B**) The cell proliferation (**A**) and migration (**B**) of SCC25-A-shNC, SCC25-A-shMETTL3, CAL27-A-shNC, and CAL27-A-shMETTL3 were tested by CCK-8 assay and transwell migration assay, respectively. (**C**) After being treated with a serial dose of cisplatin for 24 h, the cell viability of these cells was assessed by CCK-8 assay. (**D**,**F**) BALB/c strain nude mice were implanted with CAL27-A-shNC or CAL27-A-shMETTL3 and treated with cisplatin or PBS twice per week, commencing 10 days after tumor implantation. Beginning at day 7 after tumor implantation, tumor volumes were measured twice a week (D). Tumor weight was taken after all mice were sacrificed (**E**). (**F**) Using ELISA assay, the levels of TNF-α, IFN-γ, IL-2, GM-CSF, IL-10, IL-4, TGF-β, and IL-17 in tumor tissues were determined. * *p* < 0.05 compared to the control group; # *p* < 0.05 compared to the cisplatin group.

To explore the functional role of METTL3 in arecoline-transformed OSCC cells in vitro and in vivo, we established METTL3 stable knockdown OSCC cell lines using the shRNA system. We discovered that knocking down METTL3 significantly decreased cell proliferation and migration as well as sensitized cells to cisplatin-induced cytotoxicity (Figure 5A–C). These results led us to propose that targeting METTL3 might improve the tumor inhibition effect of cisplatin on arecoline-transformed OSCC. To address this question, subcutaneous injections of CAL27-A-shNC or CAL27-A-shMETTL3 were administered to nude BALB/c strain mice, followed by twice-weekly administration of cisplatin or PBS commencing 10 days post tumor implantation. The results in Figure 5D,E manifested that cisplatin treatment alone or knockdown of METTL3-alone was capable of reducing CAL27-A tumor growth.

More strikingly, the combination of shMETTL3 and cisplatin exhibited the most profound tumor inhibition effect on CAL27-A, suggesting that silencing METTL3 enhanced the therapeutical effect of cisplatin on arecoline-transformed OSCC (Figure 5D,E). As an immunosuppressive microenvironment is favorable for tumor growth and progression, we measured the levels of proinflammatory cytokines (IFN-γ, TNF-α, GM-CSF, and IL-2) and anti-inflammatory cytokines (TGF-β, IL-17, IL-10, and IL-4) within tumors. As illustrated in Figure 5F, cytotoxic cytokines (IFN-γ and TNF-α) were notably elevated, while TGF-β was markedly decreased in all three treatment groups compared to the control group (shNC + PBS). When comparing shMETTL3 and shMETTL3 + cisplatin to the control tumor, IL-2 was increased while IL-10 was decreased. Reduction of IL-17 was only observed in tumors treated with shMETTL3 + cisplatin compared to the control. Furthermore, compared to tumors treated with cisplatin alone, tumors treated with a combination of drugs had higher levels of IL-2, IFN-γ, and TNF-α while having lower levels of IL-17. No significant differences were observed in the expression levels of GM-CSF and IL-4 across the four tumor groups (Figure 5F). These results suggest that the combination of shMETTL3 and cisplatin is beneficial for creating a tumor-suppressive microenvironment by regulating the production or secretion of proinflammatory and anti-inflammatory cytokines.

## 4. Discussion

Although multiple studies have shown that METTL3 is upregulated in a variety of human malignancies, the underlying mechanisms of METTL3 dysregulation are not fully understood. In pancreatic cancer, METTL3 was overexpressed as a result of cigarette smoke condensate induced hypomethylation of the METTL3 promoter [21]. In gastric cancer, P300 was found to promote METTL3 transcription by mediating histone H3 acetylation [22]. METTL3 expression is also regulated by a number of miRNAs (e.g., miR-24-2, let-7g, and miR-186) [23,24,25]. The detailed molecular mechanism by which the METTL3 gene is regulated at the transcriptional level is largely unknown. We identified HIF-1α, a transcription factor, as being capable of binding to the upstream of the promoter region (−421/−417 bp) of METTL3 gene and increasing its expression. As our team was working on experimental confirmation of this hypothesis, Yang et al. published a similar discovery on colorectal cancer [26]. Combined together, these findings suggest that HIF-1α might be a universal transcriptional factor for METTL3 gene in a variety of human malignancies.

HIF-1α overexpression has been confirmed in a variety of human malignancies, such as bladder, breast, colon, lung, brain, oral, etc. [27]. HIF-1α elevation is associated with more aggressive phenotypes and higher patient mortality [28,29]. Recent studies have shown that areca nuts and arecoline can induce HIF-1α expression in oral fibroblasts and oral cancer cells [30]. To investigate the mechanism of arecoline-induced HIF-1α expression, we aimed to identify an arecoline-activated transcription factor that can regulate HIF-1α gene expression. MYC was chosen for further study because studies have reported that arecoline treatment markedly increases MYC activity [16] and that MYC enhances the expression levels and activity of HIF-1α via stabilizing HIF-1α in both normoxic and hypoxic conditions [17]. In addition, MYC has been identified as a target of METTL3 in acute myeloid leukemia, gastric cancer, prostate cancer, and OSCC [19]. Indeed, our results showed that MYC or METTL3 overexpression promoted HIF-1α expression. On the other hand, MYC-induced HIF-1α expression was eliminated by knockdown of METTL3. Hence, our results suggest that under the influence of arecoline, METTL3-HIF-1α-MYC establishes a positive feedback loop that promotes the progression of OSCC.

Our biological function studies illustrated that knocking down METTL3 considerably reduced cell proliferation, migration, and tumor growth of arecoline-transformed OSCC. Cisplatin, an agent with potent antitumor efficacy in clinical trials for a variety of malignancies, is the most extensively used first-line chemotherapeutic drug for OSCC [31,32]. The emergence of cisplatin resistance is a major challenge for OSCC treatment [33]. Moreover, long-term exposure to arecoline or areca nut extract has been shown to increase cisplatin or fluorouracil tolerance in OSCC cell lines and normal keratinocytes in a number of investigations [30,34]. Our previous study has shown that arecoline-transformed OSCC cell lines are more resistant to cisplatin than their parental cell lines, which supports these findings. Here, we found that silencing METTL3 sensitized arecoline-transformed OSCC to cisplatin treatment in vitro, and the combination of METTL3 knockdown and cisplatin yielded the most profound tumor inhibition effect on arecoline-transformed OSCC in vivo, suggesting that targeting METTL3 might be a wise strategy to improve therapeutic effects of cisplatin on OSCC patients, particularly in those who have a propensity of chewing areca nuts.

Although our current findings are encouraging, a few crucial issues need to be addressed in future studies. The mechanism of METTL3-mediated cisplatin sensitivity in arecoline-transformed OSCC needs to be investigated, and efficient medication delivery strategies in vivo must be developed by applying CRISPR/Cas9-mediated METTL3 gene editing technology or administering a METTL3 inhibitor. In addition, it is also strongly advised to adopt a mouse model to study the involvement of arecoline-induced METTL3 in oral submucous fibrosis transformation [35].

## 5. Conclusions

Collectively, we discovered that METTL3 was considerably elevated in OSCC of patients with areca nut chewing history and in chronic arecoline-transformed oral cancer cell lines. Our study also revealed the critical role of the METTL3/HIF-1α/MYC signaling pathway in arecoline-promoted OSCC malignancy. Arecoline-induced upregulation of MYC promotes HIF-1α expression, and HIF-1α binds the promoter region of METTL3 gene, resulting in METTL3 upregulation. The enhanced METTL3 levels, in turn, stabilize MYC mRNA via m6A modification and elevate MYC protein levels in OSCC cells. More importantly, arecoline-induced METTL3 confers multiple advantages to OSCC cells, including cell proliferation, cell migration, and cisplatin resistance, whereas these beneficial effects are diminished via depletion of METTL3. Thus, by controlling METTL3/HIF-1/MYC signaling in oral cancer, our investigations reveal a previously unidentified connection between arecoline and cisplatin resistance that may help develop strategies to overcome the challenge of cisplatin resistance in OSCC patients.

## Figures and Tables

**Table 1:** The association between METTL3 expression and clinical parameters in 66 OSCC patients.

	Relative METTL3 mRNA Levels	*p*-Value
Gender		
Female	1.83	0.061
Male	2.06	
Age		
<65	2.01	0.83
≥65	2.05	
Areca nut chewing habit		
Yes	1.75	0.041
No	2.18	
Tumor Stage		
1 and 2	1.89	0.055
3 and 4	2.01	
Nodal Stage		
0	1.83	0.073
1	1.95	
2	2.02	
Recurrence		
No	1.24	0.034
Local	1.66	
Distant	2.03	

## Data Availability

Data are contained within the article or Supplementary Materials.

## Supplementary Materials

The following supporting information can be downloaded at: https://www.mdpi.com/article/10.3390/cells11223605/s1, Figure S1: METTL3 was significantly upregulated in arecoline-induced oral cancer cell line; Table S1: Primers for RT-qPCR assay, ChIP assay, and luciferase reporter construction.
