# Supplementary material for: RNA N6-Methyladenosine (m6A) Methyltransferase-like 3 Facilitates Tumorigenesis and Cisplatin Resistance of Arecoline-Exposed Oral Carcinoma
Source: Cells. 2022 Nov 14;11(22):3605. doi: 10.3390/cells11223605 (PMC9688745; doi:10.3390/cells11223605)
Supplement: Supplementary file 1 [file cells-11-03605-s001.zip › cells-1992283-supplementary.pdf]

## Supporting Information

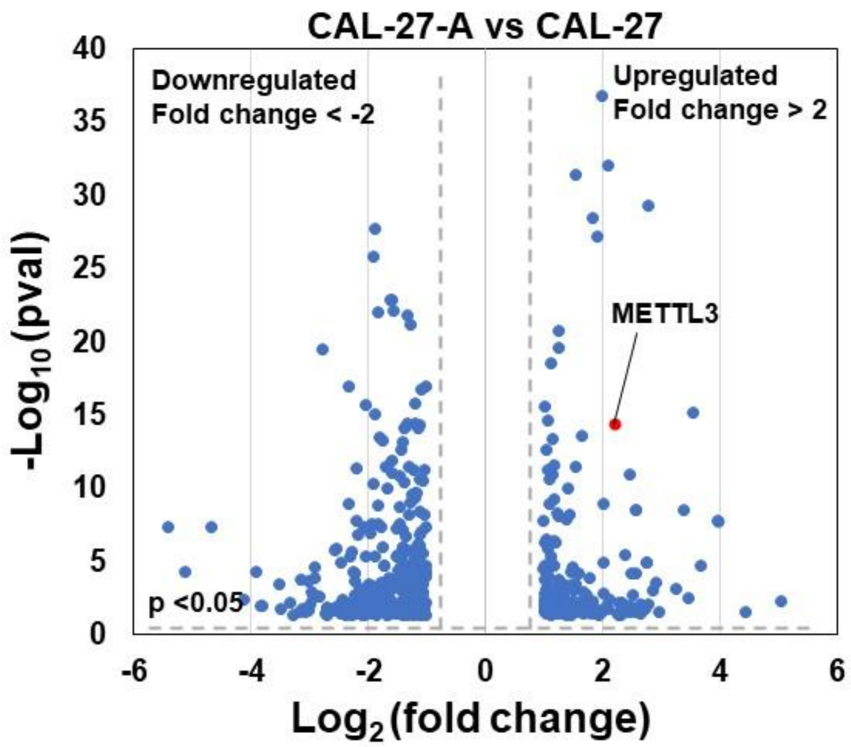


**Figure S1. METTL3 was significantly upregulated in arecoline-induced oral cancer cell line.** Volcano plot of RNA-seq transcriptome data displaying the pattern of gene expression values for CAL27-A to CAL-27. Significantly differentially expressed genes are shown ($p < 0.05$, and fold change < -2 or > 2). *METTL3* gene was highlighted in red.

**Table S1. Primers for RT-qPCR assay, ChIP assay, and Luciferase reporter construction.**

| **RT-qPCR Primers** | | |
|---|---|---|
| Gene | Forward | Reverse |
| HIF-1α | 5′-TATGAGCCAGAAGAACTTTTAGGC-3′ | 5′-CACCTCTTTTGGCAAGCATCCTG-3′ |
| METTL3 | 5′-CTATCTCCTGGCACTCGCAAGA-3′ | 5′-TGGAGGATGTGCCAGAGGTA-3′ |
| MYC | 5′-CCTGGTGCTCCATGAGGAGAC-3′ | 5′-GCTTGAACCGTGCAACCACATC-3′ |
| GAPDH | 5′-AATCCCATCACCATCTTCC-3′ | 5′-CATCACGCCACAGTTTCC-3′ |
| **ChIP assay Primers** | | |
| Gene | Forward | Reverse |
| METTL3 | 5'-GTGATTCCCGAAATGTTT-3' | 5′-TCCTGGATTCTAGCTGCC-3′ |
| **Luciferase reporter construction primers** | | |
| pGL3-METTL3 | Forward | Reverse |
| WT | 5′-GCCGGTACCGGCGTGGTGGCGGGCGCCTGTA-3′ | 5′-CGCAGATCTAGCCACGTTAGGTGTTGGCAGAG-3′ |
| MUT1 | 5′-GCCGGTACCGGCGTGGTGGCGGGCGCCTGTA-3′ | 5′-CGCAGATCTAGCTTAATTAGGTGTTGGCAGAG-3′ |
| MUT2 | 5′-GCCGGTACCGGTTAAGTGGCGGGCGCCTGTA-3′ | 5′-CGCAGATCTAGCCACGTTAGGTGTTGGCAGAG-3′ |
| MUT3 | 5′-GCCGGTACCGGTTAAGTGGCGGGCGCCTGTA-3′ | 5′-CGCAGATCTAGCTTAATTAGGTGTTGGCAGAG-3′ |

**Figure Legends:**

**Figure S1. METTL3 is upregulated in CAL27-A compared to CAL-27.** Volcano plot of RNA-seq transcriptome data displaying the pattern of gene expression values for CAL27-A to CAL-27. Significantly differentially expressed genes are shown ($p < 0.05$, and fold change $< -2$ or $> 2$).

**Table S1. Primers for RT-qPCR assay, ChIP assay, and Luciferase reporter construction**
